# Neuroplasticity and Pain Perception: Exploring the Complexities of Temporomandibular Disorders

**DOI:** 10.7759/cureus.79098

**Published:** 2025-02-16

**Authors:** Pinaki Wani, Rohit Anand

**Affiliations:** 1 Physiology, All India Institute of Medical Sciences, Raebareli, Raebareli, IND; 2 Dentistry, Private Practice, Lucknow, IND

**Keywords:** central sensitization, chronic pain, neuroimmune interactions, neuroplasticity, pain perception, peripheral sensitization, temporomandibular disorders, trigeminal nerve

## Abstract

Temporomandibular disorders (TMDs) are prevalent conditions affecting the temporomandibular joint (TMJ), masticatory muscles, and associated structures, leading to pain, restricted movement, and joint noises. These disorders are multifactorial in origin, involving structural, functional, and psychological components. This review delves into the neurophysiological mechanisms of pain perception in TMDs, focusing on peripheral and central processes, including the role of neural plasticity in chronic pain. Peripheral mechanisms involve nociceptors in the TMJ, activated by inflammatory mediators, mechanical stress, and tissue damage, leading to pain. Peripheral sensitization, driven by factors such as cytokines and neuropeptides, enhances nociceptor sensitivity, contributing to chronic pain states. The trigeminal nerve is pivotal in transmitting nociceptive information to the central nervous system (CNS), with C-fibers and A-delta fibers involved in pain perception. Central sensitization, a hallmark of chronic pain in TMDs, involves neuroplastic changes in the CNS, including wind-up and long-term potentiation (LTP), enhancing pain perception and facilitating pain persistence. Neuroplasticity, both central and peripheral, plays a critical role in the development of chronic pain. Central plasticity includes synaptic changes and alterations in brain connectivity, which were observed in functional imaging studies of TMD patients. Peripheral plasticity involves the upregulation of ion channels and neurotransmitters, sustaining pain signals. Additionally, neuroimmune interactions between microglia, astrocytes, and pain pathways are integral to central sensitization. Understanding these mechanisms is crucial for developing effective treatments targeting both peripheral and central pain processes. Emerging therapies, including transient receptor potential (TRP) channel blockers and neuroimmune modulators, offer new avenues for managing TMD pain, emphasizing the need for a multifaceted treatment approach.

## Introduction and background

Temporomandibular disorders (TMDs) encompass various conditions affecting the temporomandibular joint (TMJ), masticatory muscles, and associated structures. These disorders are characterized by pain, restricted jaw movement, and noises in the joint during mandibular function. TMDs are prevalent in the general population, affecting up to 10-15% of adults, with a higher incidence in women and individuals aged 20-40 years [1]. Despite their prevalence, TMDs present a significant diagnostic and therapeutic challenge due to their multifactorial etiology, which includes structural, functional, and psychological components. Understanding the pain mechanisms in TMDs is crucial for several reasons. First, chronic pain associated with TMDs significantly impacts patients' quality of life, leading to physical discomfort, psychological distress, and social limitations [2]. Second, effective management of TMD pain requires a comprehensive understanding of both peripheral and central pain mechanisms, including the role of neural plasticity in developing and maintaining chronic pain states [3]. Third, advancements in neurophysiological research have opened new avenues for targeted therapies that can modulate pain pathways and neural plasticity, offering hope for more effective treatments [4]. This review aims to provide a detailed examination of the neurophysiological aspects of pain perception in TMDs. We will explore the peripheral mechanisms involved, such as the role of nociceptors and peripheral sensitization, followed by an in-depth analysis of central mechanisms, including central sensitization and cortical processing of pain. Additionally, we will discuss the critical role of neural plasticity in chronic TMD pain and its implications for treatment. By integrating current research findings, this review seeks to enhance our understanding of TMD pain mechanisms and highlight potential therapeutic strategies.

## Review

Peripheral mechanisms of pain in TMDs

Role of Nociceptors in the Temporomandibular Joint

Nociceptors are specialized sensory receptors that detect noxious stimuli and initiate the pain response. In the context of temporomandibular joint disorders (TMDs), nociceptors located within the temporomandibular joint (TMJ) play a crucial role in pain perception. Various factors activate these nociceptors, including inflammatory mediators, mechanical stress, and tissue damage.

Inflammatory mediators, such as prostaglandins, bradykinin, and cytokines (e.g., IL-1β, TNF-α), contribute to the activation and sensitization of nociceptors in TMDs. Mechanical stress from occlusal abnormalities or excessive muscle contractions can also directly activate nociceptors, leading to the perception of pain [5]. Additionally, tissue damage resulting from trauma or degeneration in the TMJ exacerbates nociceptor activation. Transient receptor potential (TRP) channels, particularly TRPV1 and TRPA1, are integral to nociceptor function in the TMJ. These ion channels are activated by noxious stimuli and play a significant role in translating pain signals to the central nervous system. The altered expression and function of TRP channels in the TMJ are associated with heightened pain sensitivity and the persistence of pain in TMDs [6].

Peripheral Sensitization

Peripheral sensitization refers to the increased sensitivity of nociceptors to stimuli, which can result from inflammation, nerve injury, or prolonged pain. In TMDs, peripheral sensitization contributes significantly to the pain experience. The mechanisms underlying peripheral sensitization include the upregulation of ion channels, changes in receptor expression, and the release of inflammatory mediators. Inflammatory cytokines, such as interleukin-6 (IL-6), and neuropeptides like substance P and calcitonin gene-related peptide (CGRP) play pivotal roles in this process. These mediators not only enhance nociceptor sensitivity but also contribute to the development of chronic pain by maintaining a state of heightened inflammation in the TMJ [7]. Peripheral nerve injury, whether due to direct trauma or surgical intervention, can lead to long-lasting changes in nociceptor function and contribute to chronic pain states in TMDs. The injury can induce a cascade of events, including neuronal plasticity and alterations in neurotransmitter release, that perpetuate pain [8].

Afferent Pathways

The trigeminal nerve (cranial nerve V) is the primary sensory nerve for the TMJ, and its branches are essential for transmitting pain signals from the joint to the central nervous system. The trigeminal nerve has three major branches: the ophthalmic, maxillary, and mandibular nerves. The mandibular nerve innervates the TMJ and is involved in conveying nociceptive information. Pain signals from the TMJ are transmitted through both C-fibers and A-delta fibers. C-fibers, unmyelinated, convey dull, aching pain, while A-delta fibers, myelinated, transmit sharp, localized pain. Both types of fibers play a role in the perception of pain in TMDs. The interaction between these fibers and their central projections affects the overall pain experience and contributes to the complexity of TMD pain [9].

Central mechanisms of pain in TMDs

Central Sensitization in Chronic Pain

Central sensitization refers to the increased responsiveness of neurons in the central nervous system (CNS) to nociceptive stimuli, leading to heightened pain perception. This phenomenon is particularly relevant in chronic pain conditions, including TMDs, where pain persists beyond the expected duration of tissue healing. Central sensitization contributes to widespread pain and heightened sensitivity in affected individuals [4].

Key Mechanisms Underlying Central Sensitization

Wind-up: Wind-up is characterized by the progressive increase in neuronal excitability and pain perception with repetitive stimulation. This occurs due to the accumulation of excitatory neurotransmitters in the spinal cord, leading to prolonged activation of pain pathways [10].

Long-term potentiation (LTP): LTP is a process where repeated activation of synapses leads to a long-lasting increase in synaptic strength. In the context of pain, LTP in the spinal cord and brainstem enhances the transmission of nociceptive signals, contributing to the maintenance of chronic pain [11].

Changes in receptor expression: Central sensitization is also associated with alterations in the expression of pain-related receptors, such as NMDA receptors and glutamate receptors. These changes result in enhanced excitatory signaling and reduced inhibitory control within the CNS [12].

Role of NMDA Receptors and Glutamate in Central Sensitization

NMDA receptors, a subtype of glutamate receptors, play a crucial role in central sensitization. Activation of NMDA receptors by glutamate leads to increased intracellular calcium levels and activation of signaling pathways that promote synaptic plasticity and pain amplification. This process is essential for the development of central sensitization and the persistence of chronic pain [13].

Brainstem and Pain Processing: The Role of the Trigeminal Nucleus Caudalis in Pain Transmission

The trigeminal nucleus caudalis (TNC) is a critical relay station in the brainstem for processing nociceptive signals from the orofacial region, including the temporomandibular joint. It receives input from trigeminal afferent fibers and integrates pain signals before transmitting them to higher brain centers. The TNC plays a key role in modulating pain perception through its interactions with descending inhibitory and facilitatory pathways [14].

Integration of Sensory Input in the Brainstem and Its Modulation by Descending Pathways

In the brainstem, sensory inputs are integrated and modulated by descending pathways from the brain. These descending pathways, originating from structures such as the periaqueductal gray (PAG) and the rostral ventromedial medulla (RVM), can either inhibit or facilitate nociceptive transmission in the TNC. The balance between these modulatory influences is crucial for determining the final perception of pain [15].

Interaction Between the Trigeminal System and Other Craniofacial Pain Pathways

The trigeminal system interacts with other craniofacial pain pathways, including the cervical spinal cord and brainstem nuclei involved in facial pain. This interaction can contribute to the complexity of pain processing in TMDs, where overlapping pain signals from different sources may exacerbate pain perception. The integration of these pathways highlights the multi-faceted nature of pain in TMDs and the need for a comprehensive understanding of pain mechanisms [3].

Cortical processing of pain

Brain Regions Involved in Pain Perception

Pain perception involves several brain regions including the following [16].

Primary somatosensory cortex (S1): This is responsible for the sensory-discriminative aspects of pain such as localization and intensity.

Secondary somatosensory cortex (S2): This contributes to the processing of pain intensity and its affective components.

Insular cortex: This is involved in the emotional and cognitive aspects of pain, integrating sensory, and affective information. 

Anterior cingulate cortex (ACC): This plays a key role in the affective and motivational aspects of pain, influencing pain-related behavior and emotional responses.

Neural plasticity in TMDs

Neural plasticity refers to the ability of the nervous system to adapt structurally and functionally in response to various stimuli, including injury or chronic pain. This phenomenon includes both adaptive changes that enhance functionality and maladaptive changes that can lead to chronic pain conditions. In the context of chronic pain, neural plasticity often results in persistent pain states due to alterations in neural circuits that continue to process pain signals even after the initial insult has resolved [17]. In chronic pain conditions, such as TMDs, neural plasticity plays a pivotal role. Adaptive changes can improve sensory discrimination and pain modulation in acute situations, but maladaptive changes contribute to the development and maintenance of chronic pain. The mechanisms underlying these changes involve alterations in synaptic strength, receptor expression, and the connectivity of neural circuits within both peripheral and central nervous systems [12].

Central plasticity in TMDs

Central plasticity encompasses structural and functional alterations within the central nervous system (CNS) that are associated with chronic pain conditions like TMDs. One of the primary mechanisms involved is synaptic plasticity, which includes long-term potentiation (LTP) and changes in synaptic connectivity. These alterations contribute to enhanced pain processing and the establishment of a persistent pain state. Structural changes in the CNS, such as cortical reorganization and alterations in brain connectivity, also play a role in central plasticity. Functional neuroimaging studies have shown that patients with TMDs exhibit changes in brain regions associated with pain processing, including the primary somatosensory cortex (S1), secondary somatosensory cortex (S2), and the anterior cingulate cortex (ACC). These changes are indicative of maladaptive plasticity that contributes to the chronification of pain [17].

Peripheral plasticity in TMDs

Peripheral plasticity in TMDs involves changes in the expression and function of ion channels, receptors, and neurotransmitters within the periphery, particularly in the TMJ and surrounding tissues. Chronic stimulation or injury can lead to the upregulation of various ion channels, such as transient receptor potential (TRP) channels and ionotropic receptors, including the P2X3 receptor [18]. Additionally, peripheral plasticity is marked by increased expression of neurotransmitters and neuropeptides such as substance P and calcitonin gene-related peptide (CGRP). These changes enhance nociceptive signaling and contribute to the sustained perception of pain [19]. In TMDs, these adaptations facilitate heightened sensitivity and the persistence of pain signals even in the absence of ongoing tissue damage.

Neuroimmune interactions

The interactions between neurons and the immune system, particularly the roles of microglia and astrocytes, are crucial in central plasticity and pain modulation. In the context of TMDs, microglial activation and astrocyte involvement in the trigeminal system have been observed to contribute significantly to central sensitization. Microglia and astrocytes release pro-inflammatory cytokines and other signaling molecules that modulate pain pathways and exacerbate pain states. This neuroimmune signaling facilitates central plasticity and the maintenance of chronic pain. Targeting these neuroimmune pathways presents a promising therapeutic approach for managing TMD-related pain, with potential interventions including anti-inflammatory agents and neuroimmune modulators [20].

Implications for treatment

Effective management of TMDs requires a multifaceted approach addressing both peripheral and central mechanisms of pain, as well as strategies to modulate neural plasticity. This section discusses current and emerging treatment modalities targeting these mechanisms.

Targeting peripheral mechanisms

Pharmacological Interventions

Non-steroidal anti-inflammatory drugs (NSAIDs): NSAIDs are commonly used to manage pain and inflammation in TMDs. They work by inhibiting cyclooxygenase enzymes (COX-1 and COX-2), reducing the synthesis of prostaglandins involved in pain and inflammation. Despite their efficacy, long-term use may be limited by gastrointestinal and renal side effects [21].

Local anesthetics: Local anesthetics, such as lidocaine and bupivacaine, provide temporary relief by blocking sodium channels and preventing nociceptive signal transmission. These agents are often used in diagnostic blocks and as part of a comprehensive treatment plan [22].

TRP channel blockers: Transient receptor potential (TRP) channel blockers are an emerging class of analgesics. TRP channels, particularly TRPV1, play a significant role in pain sensation and inflammation. Inhibition of these channels has shown promise in reducing TMD-related pain [23].

Non-pharmacological Approaches

Physical therapy: Physical therapy, including exercises and manual therapy, aims to improve jaw function and alleviate pain. Techniques such as stretching, strengthening exercises, and postural training have been effective in managing TMD symptoms.

Occlusal splints: Occlusal splints or bite guards are used to reduce bruxism and jaw clenching, which are common in TMD patients. These devices help to distribute occlusal forces evenly, thereby reducing muscle strain and joint stress.

Acupuncture: Acupuncture has been explored as a treatment modality for TMDs. It is believed to modulate pain perception through the stimulation of specific points, which may influence neurotransmitter release and pain pathways [24].

Targeting central mechanisms

Pharmacological Interventions

NMDA receptor antagonists: NMDA receptor antagonists, such as ketamine, are used to modulate central sensitization and pain perception. These agents inhibit the glutamate pathway, which is implicated in chronic pain states.

Antidepressants: Tricyclic antidepressants (TCAs) and serotonin-norepinephrine reuptake inhibitors (SNRIs) are effective in treating chronic pain by modulating neurotransmitter systems involved in pain perception. They are particularly useful in managing neuropathic pain associated with TMDs.

Anticonvulsants: Anticonvulsants such as gabapentin and pregabalin are used to treat neuropathic pain by inhibiting excitatory neurotransmitter release and stabilizing neuronal activity. They have shown efficacy in reducing pain in TMD patients [21,25].

Non-pharmacological Approaches

Cognitive-behavioral therapy (CBT): CBT addresses the psychological components of chronic pain by helping patients manage stress, alter pain-related thoughts, and develop coping strategies. It has been effective in improving the quality of life and reducing pain severity in TMD patients.

Biofeedback: Biofeedback techniques teach patients to control physiological processes such as muscle tension and heart rate, which can help in managing pain. In TMD management, biofeedback has shown the potential to reduce muscle tension and pain levels.

Mindfulness: Mindfulness-based interventions, including meditation and relaxation techniques, help patients focus on the present moment and reduce pain perception. These approaches have been beneficial in reducing pain and improving function in TMD patients [26].

Modulating Neural Plasticity

The potential role of neuroplasticity-modulating agents is as follows.

Ketamine: Ketamine, an NMDA receptor antagonist, is used for its rapid antidepressant and analgesic effects. It has shown potential in modulating neural plasticity and providing relief in chronic pain conditions.

Gabapentinoids: Gabapentinoids such as gabapentin and pregabalin modulate calcium channels and neurotransmitter release, impacting central sensitization and neural plasticity. They are effective in managing chronic pain associated with TMDs.

Cannabinoids: Cannabinoids interact with the endocannabinoid system to modulate pain and inflammation. Their potential to alter neural plasticity and provide analgesic effects makes them a promising avenue for TMD pain management [27].

Neuromodulation Techniques

Transcranial magnetic stimulation (TMS): TMS involves applying magnetic fields to specific brain regions to modulate neuronal activity. It has shown promise in reducing pain perception and improving function in chronic pain conditions, including TMDs

Spinal cord stimulation (SCS): SCS involves implanting a device that delivers electrical impulses to the spinal cord to modulate pain signals. It has been effective in treating various chronic pain conditions, including TMD-related pain [28].

Emerging Therapies

Gene therapy: Gene therapy aims to correct or modify genetic abnormalities associated with pain pathways. Although still experimental, it holds the potential for addressing the underlying mechanisms of chronic pain in TMDs [29].

Stem cell therapy: Stem cell therapy seeks to repair or regenerate damaged tissues and modulate pain pathways. Research is ongoing to determine its efficacy and safety in treating TMDs [30].

Future directions

Need for Further Research into the Neurophysiological Mechanisms of TMD Pain

Despite significant advances in understanding the neurophysiological mechanisms underlying TMDs, many aspects remain poorly elucidated. Future research should focus on the detailed characterization of peripheral and central mechanisms contributing to TMD pain. For instance, more comprehensive studies are needed to explore the specific roles of various ion channels, receptors, and inflammatory mediators in peripheral sensitization and their interactions with central pain pathways. Additionally, elucidating the precise mechanisms of central sensitization and its long-term effects on pain perception could provide insights into chronic pain development. A deeper understanding of these processes could inform the development of more targeted and effective treatments [31].​​​​​​​

Potential for Personalized Medicine Approaches in TMD Management

The future of TMD management may increasingly rely on personalized medicine approaches. By integrating genetic, phenotypic, and neurophysiological data, personalized medicine aims to tailor treatments to individual patient profiles, improving efficacy and reducing adverse effects. Genetic markers related to pain perception and susceptibility to chronic pain could guide the selection of pharmacological interventions. Additionally, personalized approaches could optimize non-pharmacological treatments by considering individual responses to therapies such as physical therapy or cognitive-behavioral interventions. Emerging technologies, such as pharmacogenomics and bioinformatics, will play a crucial role in the personalization of TMD management [32].​​​​​​​

Role of Advanced Neuroimaging Techniques in Understanding TMDs

Advanced neuroimaging techniques, such as functional magnetic resonance imaging (fMRI), positron emission tomography (PET), and diffusion tensor imaging (DTI), offer promising avenues for enhancing our understanding of TMDs. These techniques can provide insights into brain activity, connectivity, and structural changes associated with TMD pain. For example, fMRI studies can reveal alterations in pain processing networks while PET imaging can help visualize changes in neurochemical activity. DTI can elucidate disruptions in white matter pathways related to pain perception and modulation. Integrating these advanced neuroimaging techniques with clinical assessments and other biomarker data will be essential for developing a more comprehensive understanding of TMDs and improving diagnostic and therapeutic strategies [33].

Implications of Neuroplasticity Research for the Development of Novel Therapies

Research into neural plasticity has profound implications for developing novel therapies for TMDs. Understanding the mechanisms of neural plasticity, including both structural and functional changes in the central nervous system, could lead to innovative therapeutic strategies aimed at modulating maladaptive plasticity. For example, therapies that target specific synaptic changes or neuroimmune interactions may help reverse or attenuate chronic pain. Techniques such as transcranial magnetic stimulation (TMS) and deep brain stimulation (DBS) hold the potential for modulating neural circuits involved in pain processing. Additionally, advances in gene therapy and regenerative medicine, such as stem cell-based approaches, offer exciting possibilities for reversing or repairing pain-associated neural changes. Continued research in neuroplasticity will be critical in advancing these therapeutic modalities and improving outcomes for patients with TMDs [34].

## Conclusions

This review highlights the complex interplay between central and peripheral mechanisms in the perception of pain associated with temporomandibular disorders (TMDs). Key findings include the pivotal roles of nociceptors in peripheral pain signaling, the impact of central sensitization on pain amplification, and the significant influence of neural plasticity on chronic pain development. A multifaceted approach to TMD pain management is essential, integrating pharmacological treatments, physical therapies, and psychological interventions to address the diverse mechanisms contributing to pain. Future research should focus on advancing our understanding of the neurophysiological underpinnings of TMDs, exploring novel therapeutic strategies targeting neural plasticity, and refining personalized treatment approaches to improve patient outcomes.
